# Comparative effectiveness and safety of sodium-glucose cotransporter 2 inhibitors vs glucagon-like peptide 1 receptor agonists in elderly patients with type 2 diabetes mellitus: a meta-analysis

**DOI:** 10.3389/fendo.2025.1486655

**Published:** 2025-08-26

**Authors:** Yao Wang, Haoming Wu, Jingxian Yang, Jun Ma, Hongling Li, Xinyu He, Yibo Xiao, Yan Pan

**Affiliations:** ^1^ Clinical Medical College & Affiliated Hospital of Chengdu University, Chengdu University, Chengdu, Sichuan, China; ^2^ College of Pharmacy, Chengdu University, Chengdu, Sichuan, China; ^3^ School of Preclinical Medicine of Chengdu University, Chengdu University, Chengdu, Sichuan, China; ^4^ West China Hospital, Sichuan University, Chengdu, Sichuan, China

**Keywords:** SGLT-2i, GLP-1RA, type 2 diabetes, elderly, meta-analysis

## Abstract

**Objective:**

This systematic review aimed to evaluate the cardiovascular effectiveness and safety of initiating sodium-glucose cotransporter 2 inhibitors (SGLT2i) in comparison to glucagon-like peptide 1 receptor agonists (GLP-1RA) among elderly patients with diabetes.

**Methods:**

A comprehensive search of the PubMed, Embase, and Web of Science databases was conducted up to March 2024. The summary standard mean differences and odds ratios were calculated.

**Results:**

Twelve studies of eleven articles were included in the analysis. Older patients receiving SGLT2i had a greater incidence of euglycemic ketoacidosis (EKA) (OR 1.622, 95% CI 1.276-2.062, p = 0.000) and genitourinary infection (GUI) (OR 3.59, 95% CI 3.31-3.89, p = 0.00) than did those receiving GLP-1RA, and the opposite was true for acute kidney injury (AKI) (OR 0.902, 95% CI 0.854 - 0.952, p = 0.00). However, no significant differences were detected for major adverse cardiovascular events (MACE) (OR 1.04, 95% CI 0.95-1.13, p = 0.386), hospitalization for heart failure (HHF) (OR 0.98 95% CI 0.83-1.16, p = 0.825), myocardial infarction (MI) (OR 1.09, 95% CI 0.94-1.26, p = 0.265), stroke (OR 1.22, 95% CI 1.02-1.45, p = 0.028), total adverse events (AEs), (OR 0.98, 95% CI 0.83-1.16, p = 0.825), serious AEs (OR 1.02, 95% CI 0.94 -1.11, p = 0.594), fractures (OR 1.07, 95% CI 0.92-1.24, p = 0.394) or hypoglycemia (OR 0.95, 95% CI 0.88-1.02, p = 0.141).

**Conclusion:**

In conclusion, although SGLT2i increase the risk of EKA and GUI and GLP-1RA decrease the risk of AKI, SGLT2i are at comparable risk of MACE, HHF, MI, stroke, hypoglycemia, and fracture to GLP-1RA.

**Systematic review registration:**

https://www.crd.york.ac.uk/PROSPERO/, identifier CRD42024518348.

## Introduction

Type 2 diabetes (T2D) in older adults is a significant and growing public health challenge (1). Older adults with T2D have a high risk of microvascular and cardiovascular complications, hypoglycemia, and mortality, and this risk increases significantly as they age. Given the growing burden of diabetes, it is crucial to identify treatments that can reduce the risk of complications. Cardiovascular events significantly contribute to the morbidity and mortality of older individuals with T2D (2). Hence, a critical focus of diabetes management is the optimization of cardiovascular morbidity and mortality. Since the introduction of glucagon-like peptide-1 receptor agonists (GLP-1RA) in 2005 and sodium-glucose cotransporter-2 inhibitors (SGLT2i) in 2012, these has been a recent shift in the treatment paradigm for T2D (3, 4). In the recent large cardiovascular outcomes trials (CVOT), SGLT2i and GLP-1RA were superior to placebo in reducing the risk of major adverse cardiovascular events (MACE) (5–8), cardiovascular mortality (5), all-cause mortality (5, 9), and the progression of nephropathy (6, 8, 10, 11). However, their impact on hospitalization for heart failure (HHF) has not been fully elucidated (6, 8, 9).

Accordingly, the ADA and AACE guidelines recommend the initiation of an SGLT2i or a GLP-1RA among patients with high cardiovascular risk or patients with established atherosclerotic cardiovascular disease, heart failure, or chronic kidney disease (12, 13). Despite the promise of these newer agents, their comparative efficacy in older patients >65 years of age remains largely unknown. Several factors contribute to this issue. First, CVOT did not perform head-to-head comparisons of these drugs. Second, these trials are typically conducted in specific populations with an average age of less than 65 years. Finally, older adults with significant comorbidities, functional impairments, or limited life expectancy are explicitly excluded from CVOTs (14, 15).

As information is rapidly accumulating about potential unintentional injuries from SGLT2i and GLP-1RA, such as euglycemic ketoacidosis (EKA), acute kidney injury (AKI), fractures, genitourinary infections (GUI), gallbladder disease and volume deprivation (16, 17), it is critical to understand the safety of these medications in older patients with diabetes. Older patients are more prone to common geriatric syndromes such as accelerated muscle loss, frailty, multiple comorbidities, polypharmacy, functional decline, decreased mobility, and cognitive deficits than younger patients and are therefore at greater risk for drug-related adverse events (8, 18, 19). Thus, the risk of medication adverse events is especially important when deciding to initiate these agents in older adults.

The primary aim of this meta-analysis was to assess the cardiovascular effectiveness of initiating SGLT2i compared to GLP-1RA in elderly person with diabetes. The secondary goal was to evaluate the safety of SGLT2i versus GLP-1RA in older patients.

## Materials and methods

### Search strategy

Our study was carried out based on the preset protocol registered with CRD42024518348. The PubMed, Embase, and Web of Science were searched for literature published before March 2024 using the following keywords: “sodium-glucose cotransporter 2 inhibitor”, “SGLT2 inhibitor”, “SGLT2i”, “individual names of SGLT2 inhibitor”, “glucagon-like peptide-1 receptor agonists”, “GLP-1RA”, “individual names of GLP-1RA”, “old”, “elderly”, “type 2 diabetes”, and “T2D”. There was no language restriction on our searches. All identified articles were manually searched.

### Selection of articles

**Figure 1:**
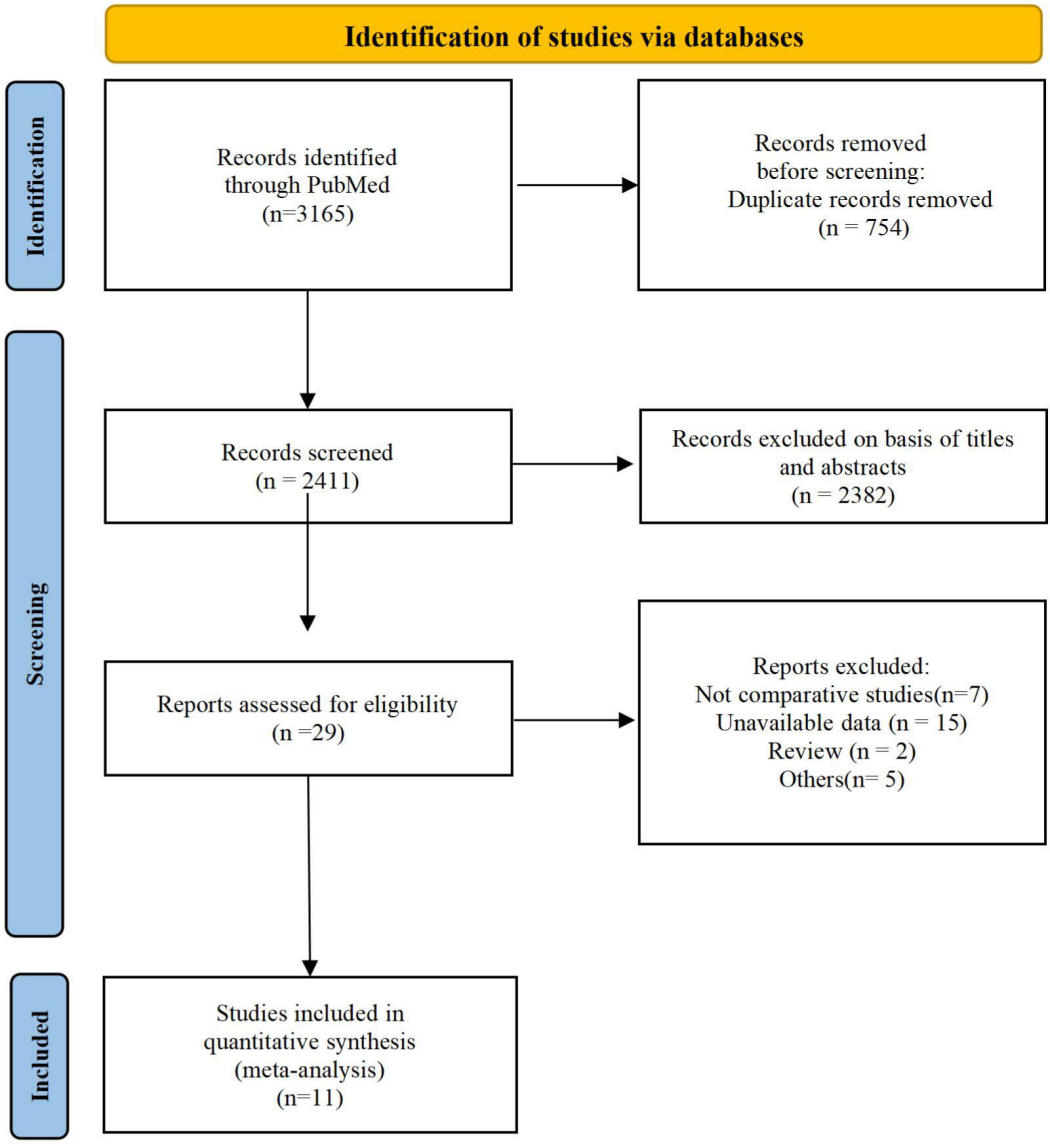
Identification of eligible articles.

We screened articles according to the following criteria. (1) The subjects were elderly patients (≥65) with T2D. (2) The study was designed as a retrospective or prospective controlled clinical trial. (3) Patients in the experimental group received SGLT2i, and those in the control group received GLP-1RA. (4) The article provided information such as complications, features, number of subjects and clinical outcomes. Studies were excluded from our meta-analysis for the following reasons: duplicate articles, unavailable data, only abstracts available, and nonclinical publications. The screening process is shown in the following diagram (**Figure 1**).

### Data extraction

For the included studies, two investigators independently extracted the following information from each study: author name, publication year and country, number of participants, mean age, participant baseline characteristics, SGLT2i types, GLP-1RA types, and clinical outcomes, including MACE, HHF, myocardial infarction (MI), stroke, adverse events (AEs), serious AEs (SAEs), fractures, AKI, hypoglycemia, EKA and GUI. Disagreements between the two investigators were resolved by a third investigator. Moreover, investigators could contact the corresponding authors of studies to obtain more information if important data were unavailable or absent.

### Statistical analysis and quality assessment

The meta-analysis was carried out based on the guidelines of the preferred reporting items for systematic reviews and meta-analyses (PRISMA) statements (16). All the statistical analyses were carried out with Stata 16.0 (Stata Corp, College Station, TX, USA). Odds ratios (ORs) with 95% confidence intervals (CIs) were calculated for dichotomous variables. The inconsistency test (I^2^) was used to assess heterogeneity when I^2^ >50% was considered high heterogeneity. If I^2^ was <50%, a fixed effects model was adopted, otherwise, a random effects model was used. We used sensitivity analyses to identify possible sources of heterogeneity. p < 0.05 was regarded as statistically significant. Publication bias was estimated by funnel plots. The Newcastle-Ottawa scale (NOS) was used for the assessment of retrospective studies, and studies of low, intermediate, and high quality were defined as those with NOS scores of 1-3, 4-6, and 7-9, respectively. When disagreements occurred, a consensus was reached with another member.

## Results

### Screening and patient characteristics

A total of 3165 studies were identified in our initial screening, 31 of which underwent full-text review. Finally, twelve studies of eleven articles (15, 20–29) were included in our meta-analysis (**Figure 1**). **Table 1** shows the baseline demographics of the included studies, while **Table 2** shows the clinical results. A total of 380527 patients received SGLT2i, and 383495 patients received GLP-1RA. The results of the quality assessment of all included studies were satisfactory and are shown in **Supplementary Table 1**. No evidence of publication bias was observed, as confirmed by the funnel plot displayed in **Supplementary Table 2**.

**Table 1:** Basic characteristics and clinical results of included studies.

Article	Year	Country	Treatments	Sample size	Age(y)
Htoo PT (20)	2022	USA	SGLT2i/GLP-1RA	11830/10142	>65
Htoo PT (21)	2023	USA	SGLT2i/GLP-1RA	82994/82994	>65
Kutz A (22)	2023	USA	SGLT2i/GLP-1RA	89865/89865	>65
Patorno E (14)	2021	USA	SGLT2i/GLP-1RA	45047/45047	>65
Thomsen RW (23)	2021	Denmark	Empagliflozin/Litaglutide	6114/4231	>65
Varshney N (24)	2021	USA	SGLT2i/GLP-1RA	133/341	>65
Xie Y (25)	2020	USA	SGLT2i/GLP-1RA	18544/23711	>65
Yang JY (26)	2021	USA	SGLT2i/GLP-1RA	8579/9765	>65
Yamada Y PIONEER 9 (27)	2021	Japan	Semaglutide/Litaglutide	21/15	>65
Yamada Y PIONEER 10 (27)	2021	Japan	Semaglutide/Dulaglutide	34/18	>65
Zhuo M (28)	2023	USA	SGLT2i/GLP-1RA	45889/45889	>65
Zhuo M (29)	2022	USA	SGLT2i/GLP-1RA	71477/71477	>65

Values are all given as SGLT2i/GLP-1RA group; USA, the United States of America.

**Table 2:** Comparison of outcomes between SGLT2i and GLP-1RA groups.

Article	MACE	HHF	MI	Stroke	AEs	SAEs	Fractures	AKI	Hypoglycemia	EKA	GUI
Htoo PT (20)	242/185	57/95	81/64	66/36	561/478	115/98	NR	NR	NR	NR	NR
Htoo PT (21)	NR	NR	NR	NR	NR	NR	NR	NR	848/898	NR	NR
Kutz A (22)	2627/2839	NR	NR	NR	2944/3223	NR	NR	NR	NR	NR	NR
Patorno E (14)	597/553	234/309	301/277	214/187	NR	310/293	181/175	1268/1352	NR	79/50	2623/753
Thomsen RW (23)	388/292	281/219	NR	NR	NR	NR	NR	NR	NR	NR	NR
Varshney N (24)	NR	NR	NR	NR	NR	NR	NR	NR	NR	NR	5/22
Xie Y (25)	NR	NR	NR	NR	NR	604/765	NR	NR	NR	NR	NR
Yang JY (26)	NR	NR	NR	NR	NR	13/46	NR	NR	NR	NR	NR
Yamada Y PIONEER 9 (27)	NR	NR	NR	NR	17/7	2/0	NR	NR	NR	NR	NR
Yamada Y PIONEER 10 (27)	NR	NR	NR	NR	32/15	NR	NR	NR	NR	NR	NR
Zhuo M (28)	NR	280/379	NR	NR	NR	NR	158/148	NR	529/557	96/58	NR
Zhuo M (29)	NR	NR	NR	NR	NR	NR	NR	1254/1438	NR	NR	NR

Values are all given as SGLT2i/GLP-1RA group; NR, not report; MACE, major adverse cardiovascular events, HHF, hospitalization for heart failure, MI, myocardial infarction, AEs, adverse events; SAEs, serious adverse events; AKI, acute kidney injury; EKA, euglycemic ketoacidosis; GUI, genitourinary infections.

### Outcomes

#### MACE

**Figure 2:**
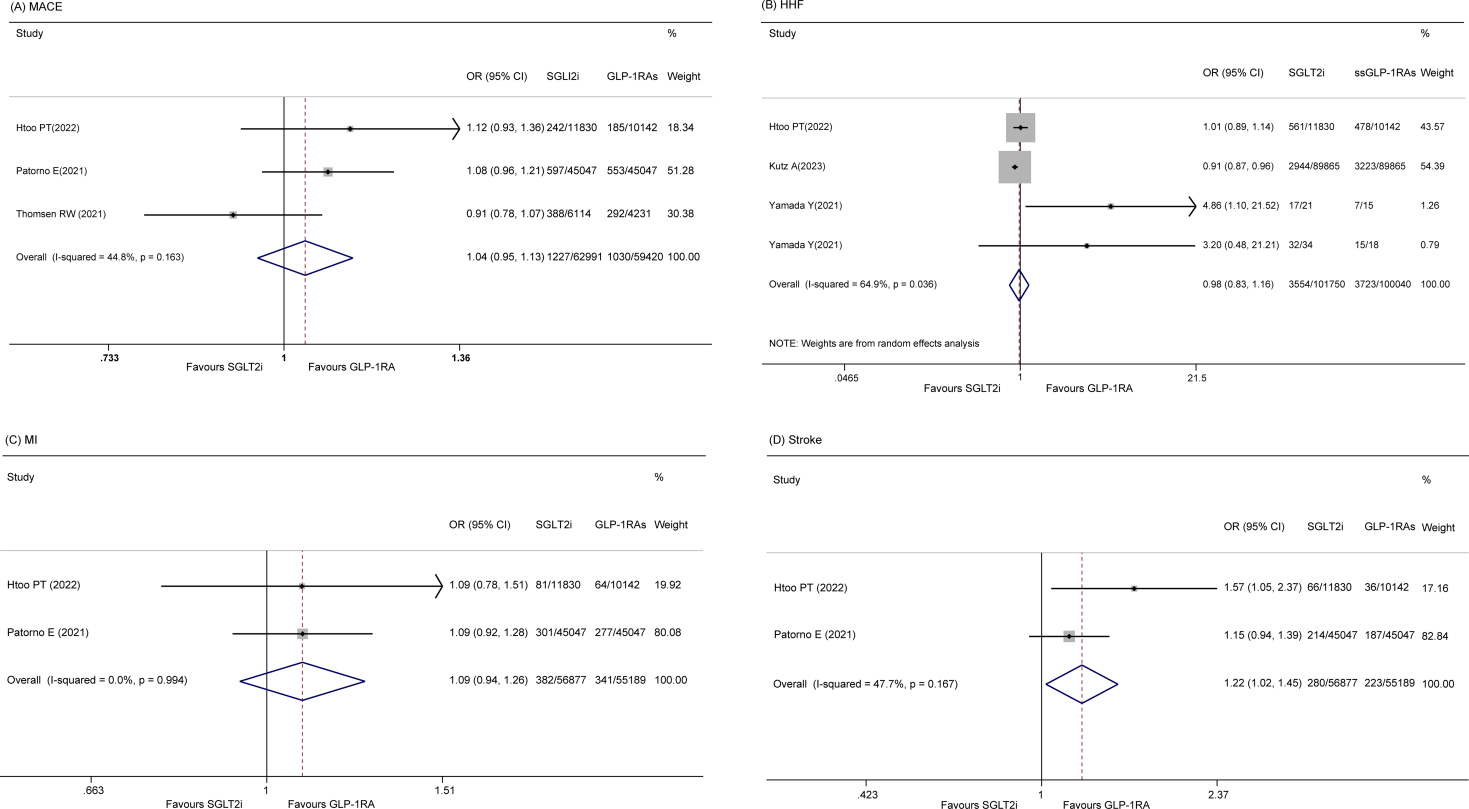
Forest plot of **(A)** MACE **(B)** HHF **(C)** MI **(D)** Stroke.

Four studies (14, 20, 22, 23) reported on the incidence of MACE. A random-effects model revealed that there was no significant difference in MACE between the SGLT2i and GLP-1RA groups (OR 0.99, 95% CI 0.90-1.10, p = 0.884; I^2^ = 66.2%, p = 0.031). However, a sensitivity analysis was performed because of the significant p value indicating heterogeneity, which showed that a study by Kutz A et al. (23) influenced the results (**Supplementary Table 3**). After removing this study, the pooled OR was 1.04 (95% CI 0.95-1.13, p = 0.386; I^2^ = 44.8%) (**Figure 2A**).

#### HHF

Four studies (20, 22, 27) discussed HHF. No significant difference between the two groups was detected (OR 0.98 95% CI 0.83-1.16, p = 0.825), and the heterogeneity was high among these studies (I^2^ = 64.9%, p = 0.036) (**Figure 2B**). However, sensitivity did not reveal the sources of the significant heterogeneity (data not shown).

#### MI

Two articles (14, 22) reported the risk of MI, and there was no statistically significant difference between the two groups (OR 1.09, 95% CI 0.94-1.26, p=0.265; I^2^ = 0.0%) (**Figure 2C**).

#### Stroke

Two studies (14, 22) reported the incidence of stroke, and no significant difference between the two groups was found (OR 1.22, 95% CI 1.02-1.45, p = 0.028; I^2^ = 47.7%) (**Figure 2D**).

#### AEs

**Figure 3:**
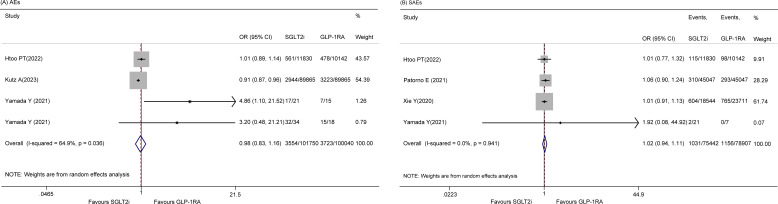
Forest plot of **(A)** AEs **(B)** SAEs.

There were four studies (20, 22, 27) on the incidence of total AEs, and a random effects model was used, indicating that there was no significant difference between the two groups (OR 0.98, 95% CI 0.83-1.16, p = 0.825; I^2^ = 64.9%) (**Figure 3A**). Sensitivity analysis revealed no sources of significant heterogeneity (data not shown). In addition, five studies (14, 20, 25–27) reported SAEs, and the results indicated that there was no significant difference between the two groups (OR 0.92, 95% CI 0.74 - 1.15, p = 0.463; I^2^ = 71.0%). However, the sensitivity analysis revealed that the study by Yang JY et al. (26) was the cause of heterogeneity. After omitting this study, the pooled OR was 1.02 (95% CI 0.94-1.11, p = 0.594; I^2^ = 0.0%) (**Figure 3B**).

#### Fractures

**Figure 4:**
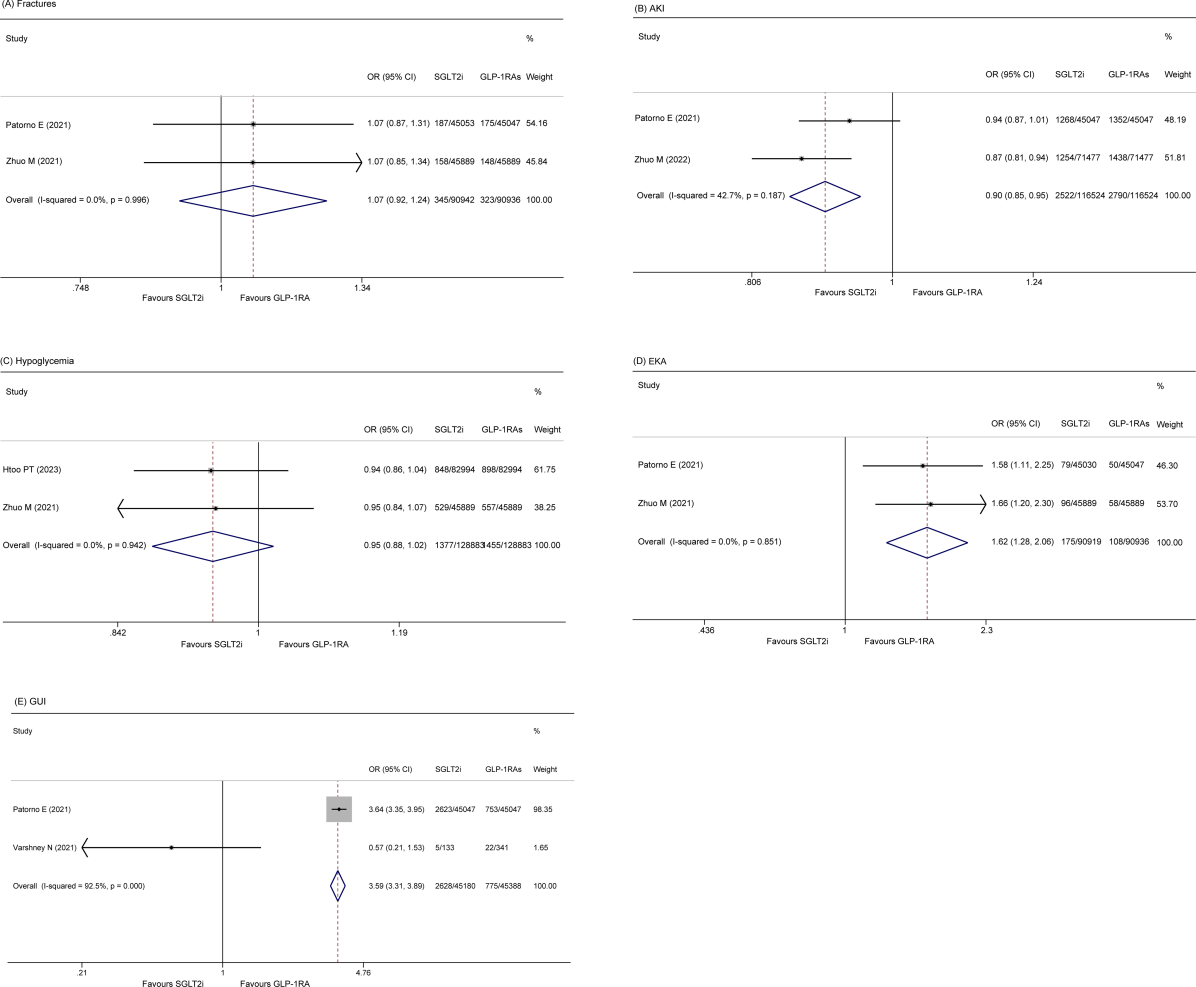
Forest plot of **(A)** Fractures **(B)** AKI **(C)** Hypoglycemia **(D)** EKA **(E)** GUI.

Two included studies (14, 28) reported the incidence of fractures among patients. A fixed effect model showed that there was no significant difference in the rate of fracture (OR 1.07, 95% CI 0.92-1.24; p = 0.394) between the two groups. The heterogeneity of these studies was low (I^2^ = 0.0%, p = 0.10) (**Figure 4A**).

#### AKI

Two studies (14, 29) reported the rate of AKI. The pooled data also revealed that elderly patients who received GLP-1RA had a greater incidence of severe AKI than those who received SLGT2i (OR 0.902, 95% CI 0.854 - 0.952, p = 0.00; I^2^ = 42.7%) (**Figure 4B**).

#### Hypoglycemia

Two studies (21, 28) included in the analysis reported the occurrence of hypoglycemia in patients. The fixed-effects model indicated no significant difference in the occurrence of hypoglycemia between the two groups (OR 0.95, 95% CI 0.88-1.02, p = 0.141). The studies exhibited low heterogeneity (I^2^ = 0.0%, p = 0.942) (**Figure 4C**).

#### EKA

Two studies (14, 28) reported EKA, and the results indicated that the SGLT2i group had a greater incidence of EKA than did the GLP-1RA group (OR 1.622, 95% CI 1.276-2.062, p = 0.000; I^2^ = 0.0%, p = 0.851) (**Figure 4D**).

#### GUI

Two studies (14, 24) reported GUI. The pooled results showed that the SGLT2i group had an increased risk of GUI relative to the GLP-1RA group (OR 3.59, 95% CI 3.31-3.89, p = 0.00; I^2^ = 92.5%) (**Figure 4E**).

## Discussion

This meta-analysis represents the first investigation comparing the effectiveness and safety of SGLT2i with GLP-1RA in elderly patients with diabetes. Our study revealed comparable risks of MACE, HHF, MI, stroke, hypoglycemia and fracture between SGLT2i and GLP-1RA. SGLT2i initiators were associated with an elevated risk of EKA or GUI and a reduced risk of AKI.

Among older adults, those taking SGLT2i had similar MACE, HHF, MI and stroke risk rates to those taking GLP-1RA in our study. However, the results of other studies of SGLT2i and GLP-1RA in older person with diabetes are not consistent with our findings. Patorno E et al. (14) showed that in patients with a history of cardiovascular disease (CVD), SGLT2i reduced the risk of developing HHF compared with GLP-1RA, whereas in patients without a history of CVD, the use of SGLT2i reduced HHF compared with GLP-1RA but with a much lower degree of benefit. However, Htoo PT et al. (20) estimated the beneficial effects of SGLT2i over GLP-1RA for HHF outcomes in all subgroups and for MACE and mortality among those with a history of both CVD and HHF. Furthermore, GLP-1RA were more favorable than SGLT2i for MACE outcomes and stroke and to a lesser extent for MI and mortality in those without documented CVD or HHF. The results of the study by Thomsen RW et al. (23) were similar to ours, they showed that empagliflozin and liraglutide had comparable rates of MACE and HHF, whereas empagliflozin initiators had a lower rate of a first HHF or loop-diuretic initiation. In the future, more head-to-head studies of SGLT2i and GLP-1RA in older person with diabetes are needed to guide our drug choices in the clinic.

Regarding safety, the incidences of total AEs and SAEs were similar between the two groups. Fractures are more common in elderly people. In the present study, there was no difference in the risk of fractures between SGLT-2i users and GLP-1RA users. SGLT-2i augments urinary phosphate reabsorption and triggers parathyroid hormone, and this action has the potential to negatively affect bone health (30, 31). Conversely, it has been postulated that GLP-1RA might have beneficial effects on bone health by promoting osteoblast differentiation and inhibiting osteoclast activity (32, 33). There is a concern that SGLT-2i may be associated with an increased risk of fracture. Therefore, the present study sought to determine whether taking SGLT-2i or GLP-1RA is associated with an increased risk of fracture in older adults. Although the effects of SGLT-2i on bone health are biologically plausible, clinical studies on fracture risk are inconsistent. According to the Canagliflozin Cardiovascular Assessment Study (CANVAS), the incidence of bone fractures among those taking canagliflozin was significantly greater than that among those taking a placebo (16). This increased fractures risk was not observed in the following large randomized controlled trials (RCTs), nor was an association observed in subsequent meta-analyses (10, 34–37). However, there was a lack of focus on elderly individuals in these studies, and therefore, there is a lack of data on the incidence of fractures in elderly individuals taking any SGLT-2i. Previous studies have shown that the use of SGLT-2i is not associated with an increased risk of fracture compared to GLP-1RA in a relatively young population (38, 39). Our analysis of studies in elderly patients led to consistent conclusions. However, more RCTs need to be conducted to confirm our findings.

Despite the renoprotective effects of long-term treatment, there is an acute decrease in the glomerular filtration rate with SGLT-2i initiation, and SGLT-2i may lead to AKI due to hypovolemia, an excessive decrease in transglomerular pressure through tubuloglomerular feedback, uricosuric action, and renal medullary hypoxia (40–42). In contrast, RCTs have shown that the incidence of AKI in SGLT-2i-treated patients does not increase (35, 43, 44) and may even be attenuated (10, 11) compared to that in patients receiving placebo, and a recent network meta-analysis of RCTs indicated that SGLT-2i may have a lower AKI risk than GLP-1RA (45). To date, the mechanism through which SGLT-2i could prevent AKI is still under investigation. In addition to potential AKI protection through heart failure and CKD risk reduction (10, 11, 43, 44, 46) it has been postulated that SGLT-2i reduces sodium and glucose reabsorption in the proximal tubule, which may lead to reduced oxygen consumption and increased resistance to ischemia perfusion injury (41). As SGLT-2i increases sodium delivery to the macula densa, it can decrease intraglomerular pressure and reduce podocyte stress through tubuloglomerular feedback (47). Furthermore, SGLT-2i could increase renal hypoxia-inducible factor expression, erythropoietin production, the suppression of peritubular inflammation and fibrosis, and the increased use of ketone bodies as an alternative fuel source (48, 49). Older age is also a significant risk factor for AKI (50). Therefore, a glucose-lowering medication reducing the risk of AKI would be advantageous for older adults. Our study provides support for the safety of SGLT-2i with respect to the risk of AKI and suggests that SGLT-2i may actually prevent AKI events compared to GLP-1RA.

Although GLP-1RA significantly reduced the risk of comorbid renal endpoints, including new-onset proteinuria and persistently elevated eGFR, the use of semaglutide was associated with a greater risk of AKI than placebo in clinical trials of GLP-1RA (51, 52). In addition, nearly 80 postmarketing reports of exenatide have shown that patients develop acute renal failure or renal insufficiency after the drug is administered (53), with 95% of these cases accompanied by renal risk factors, including the use of nephrotoxic drugs, hypertension, and heart failure. AKI (53), interstitial nephritis, and acute tubular necrosis (54) were also reported in some cases when liraglutide and semaglutide were first marketed. Renal function did not fully recover after discontinuation of the drug in these patients, and renal function and urinary protein did not improve. However, some reports suggest that GLP-1RA associated AKI may be caused by gastrointestinal reactions leading to decreased fluid intake and massive fluid loss (53). However, with numerous GLP-1RA on the market, clinical observations have revealed that not all cases can be attributed to prerenal problems (54, 55). An example is interstitial nephritis (56), which was also confirmed by semaglutide (54). The risk of AKI associated with GLP-1RA is not significantly different from that associated with other common adverse effects, possibly because AKI is a rare adverse effect, because of the limited observation period of the clinical trials, and because adverse effects are easily masked by the rapid progression of diabetic nephropathy (DKD), which makes it more likely that they will go unnoticed. Currently, most of the warning information about GLP-1RA comes from clinical trials or case reports, and only a limited number of pharmacovigilance studies have explored other adverse effects of this class of drugs (57, 58). No studies have systematically analyzed the risk and characteristics of GLP-1RA associated AKI, and there is a gap in the clinical profile of this disease in the real world. Therefore, more studies are needed to assess the association between GLP-1RA and AKI. With the increasing use of GLP-1RA in diabetes treatment, GLP-1RA induced AKI should be treated with caution. When choosing GLP-1RA as a glucose-lowering regimen, special attention should be given to patients with a high risk of nephropathy and enhanced monitoring.

Hypoglycemia is one of the most common AEs related to glucose-lowering medications in person with diabetes (59). It is a leading cause of hospital admissions and emergency department visits in older adults aged 65 years and older (59, 60). The risk of hypoglycemia is a critical consideration in the management of T2D. GLP-1 receptor agonists lower blood glucose in a glucose-dependent manner, while SGLT2i do not directly stimulate insulin secretion. Therefore, both of these newer glucose-lowering medications, which have a lower risk of inducing hypoglycemia, are particularly recommended for elderly patients (61). However, the newer agents can have an increased risk of hypoglycemia when used in combination with sulfonylureas and insulin (62). Evidence on the risk of hypoglycemia with glucose-lowering drugs comes mainly from RCTs. To date, few trials have directly compared the risk between classes of novel glucose-lowering drugs. To address these gaps in the evidence, we compared the risk of hypoglycemia in older patients between SGLT2i and GLP-1RA. The present study showed that the risk of hypoglycemia is comparable between SGLT2i and GLP-1RA. In contrast, one of the included studies by Patorno E et al. (14) reported that SGLT2i had a lower risk of hypoglycemia than GLP-1RA, with larger associations in patients using baseline insulin or sulfonylurea. They hypothesized that SGLT2i could induce glucagon release from pancreatic alpha islet cells, which could ameliorate the risk of severe hypoglycemia (63). Certainly, more head-to-head studies are needed to directly compare the risks between SGLT2i and GLP-1RA.

In the present study, the incidence of EKA was greater in the SGLT2i group than in the GLP-1RA group in elderly diabetes patients. Several factors could explain these results. First, SGLT2i reduce blood glucose levels by increasing urinary glucose excretion, thereby decreasing insulin secretion from pancreatic β-cells. Decreased circulating insulin levels lead to a reduction in the antilipolytic activity of insulin, which stimulates the production of free fatty acids, which are converted to ketone bodies by β-oxidation in the liver. The use of SGLT2i stimulates glucagon secretion, which may be a secondary effect mediated by reduced insulin secretion or a direct effect of SGLT2i on pancreatic α-cells. Second, SGLT2i may promote ketone body reabsorption by increasing the sodium concentration in renal tubules. Finally, SGLT2i have a natriuretic and osmotic diuretic effect due to the inhibition of sodium-glucose cotransporter proteins, especially in patients with high blood glucose levels, whose renally filtered glucose levels exceed the maximum limit of tubular glucose reabsorption, and whose urinary glucose levels increase, leading to osmotic diuresis. The loss of sodium ions and the osmotic diuretic effect reduce blood volume, thereby promoting ketoacidosis (64–66). Not all individuals taking SGLT2i are at high risk for EKA. Studies have reported that risk factors for the development of EKA include organic pancreatic insufficiency, pancreatic cancer, a low-carbohydrate diet, prolonged starvation, carbohydrate restriction, and the discontinuation of insulin or insulinotropic hormones at the time of initiation of treatment with SGLT2i, which are associated with a keto metabolic state induced by reduced circulating insulin levels. Therefore, caution should be exercised when prescribing SGLT2i, as well as when prescribing medications and dietary education. In patients with β-cell insufficiency, especially those with a long history of diabetes, more caution may be needed when using SGLT2i, as it is thought that β-cell function declines with age in T2D patients. Therefore, their use in elderly patients needs to be carefully considered.

It is worth emphasizing that, based on clinical symptoms alone, EKA can easily be missed because it is not necessarily associated with the typical presentation of diabetic Ketoacidosis (DKA) (e.g., dehydration due to marked hyperglycemia). However, severe metabolic acidosis alone has the potential to become a life-threatening disease. Further insight into the metabolic and humoral effects of SGLT2i and more detailed clinical information on associated cases of EKA could help to provide a stronger foundation for the safe, appropriate, and widespread use of such new drugs.

It is well known that the risk of GUI is generally increased in patients with diabetes due to the availability of glucose in the uroepithelium and changes in immune function. This risk is increased in people on SGLT2i due to increased glucosuria (67). Additionally, advanced age is an independent risk factor for GUI occurrence, which may confound the role of SGLT2i medications in causing GUI (68). The pooled results of our study showed that older patients treated with SGLT2i had a greater risk of GUI than older patients treated with GLP-1RA. The reason for the increased risk of GUI with SGLT2i is that glucose may serve as a substrate or nutritional factor, and UGEs can promote fungal growth on genital tissues (69). The risk associated with SGLT2i and GUI has varied across trials. One meta-analysis and two cohort studies reported an increased risk of GUI (70, 71). In contrast, Varshney N et al. concluded that SGLT2i use was associated with an increased amount of glycosuria and risk of genital fungal infections and that the risk of GUI may not limit the use of SGLT2i medications in appropriately selected older adults (24). Overall, older age, uncontrolled diabetes mellitus, female sex, increased BMI, CKD, and nonwhite ethnicity were considered independent risk factors for GUI (72). This may lead to caution in the use of SGLT2i initiation in these populations. More research is needed in the future to focus on GUI in older person with diabetes receiving SGLT2i.

One in every four older adults with T2D has frailty, which refers to a clinically detectable state of decreased physiological reserve and increased vulnerability to stressors and poor clinical outcomes (73). T2D increases the risk of frailty by affecting sarcopenia, mobility, cognitive impairment, and exhaustion or through microvascular and macrovascular complications, such as neuropathy or cardiovascular dysautonomia (74). Because of the greater risk of hypoglycemia and lower life expectancy, guidelines recommend less tight glycated hemoglobin targets among older and frail people with diabetes (75). However, it is still debated how these recommendations should be applied and whether certain antidiabetic medications are more favorable than others. Many clinical trials have not focused on older and frail participants with T2D, so knowledge of the efficacy and safety of new antidiabetic drugs in these clinically complex populations is lacking. Elderly patients who are frail have a significantly greater risk of hypoglycemia, fractures and GUI. Kutz A et al. reported that frailer people experienced greater benefits from SGLT-2i or GLP-1RA treatment than those without frailty (22). It is therefore of great interest to develop an appropriate strategy to balance the pros and cons of using SGLT-2i or GLP-1RA in frail patients. In response to this question, it has been proposed that frail elderly person with diabetes can be categorized into two distinct metabolic ‘phenotypes’, the anorexic malnutrition (AM) frailty phenotype and the sarcopenic obesity (SO) frailty phenotype. The AM frailty phenotype is characterized by substantial muscle loss and reduced insulin resistance. In contrast, the SO frailty phenotype is characterized by increased visceral fat and insulin resistance. Currently, there are no hypoglycemic agents specifically designed for older people with both diabetes and frailty. Therefore, Sinclair AJ et al. favor a pragmatic approach that targets the SO phenotype and favors the use of SGLT-2i or GLP-1RA to promote weight loss in the SO phenotype. SGLT-2i or GLP-1RA are used cautiously in elderly patients with an AM phenotype to reduce the risk of hypotension, dehydration, weight loss and falls, fractures, and hypoglycemia in this vulnerable group (75).

With an increasing number of older adults with T2D requiring surgery, ensuring the safety of perioperative and periprocedural management has become a critical consideration for person treated with SGLT2i and GLP-1RA. Delayed gastric emptying (GE) is associated with retained gastric contents (RGC), which can increase the risk of perioperative or periprocedural aspiration (76). Delayed GE is prevalent in people living with T2D, and old age is also a factor in the risk of RGC and pulmonary aspiration. GLP-1RA-associated GE delays may lead to RGC, which can exacerbate the risk of perioperative pulmonary aspiration. Current recommendations suggest that liquid diet the day prior to procedures likely reduces risk of RGC and a withholding period of more than 3 half-lives for GLP-1RAs with a prolonged half-life is likely more efficacious than a one-week withholding period. However, the body of evidence for what may be the best periprocedural management approach for GLP-1RA is generally weak, predominantly due to observational study designs and absence of information (77). More research support is therefore needed.

EKA is a known side effect of SGLT2i that has implications for perioperative and periprocedural management, given that surgical stress along with reduced oral intake or fasting are triggers for SGLT2i-associated ketoacidosis (78). In the May 2023 Alert Update, a multisociety group from Australia and New Zealand recommended that SGLT2i should be omitted for bowel-prepared surgeries and operations that occur ≥3 days prior to surgery and that require ≥1 day(s) of hospitalization or that require carbohydrate restriction. They also recommended that, for ambulatory surgical procedures that do not require bowel preparation, SGLT2i medications can be discontinued the same day as the surgery (i.e., in the not more than a few days prior to surgery); for those patients who do not hold SGLT2i medications as recommended, a perioperative ketosis and acidosis monitoring strategy is recommended. There exists a notable deficiency in safety comparisons regarding perioperative management of these two novel classes of hypoglycemic agents in older individuals with T2D. Consequently, further research is necessary to focus on perioperative management strategies for older individuals with T2D receiving GLP-1RAs and SGLT2i.

Our meta-analysis has several limitations. Firstly, all of the studies included were from wealthy nations such as the United States and Europe, which led to a lack of representation. Secondly, most of the studies were observational in design. Then, selection and confounding bias might exist. Finally, there are currently both SGLT2i that can be used in combination with dipeptidyl peptidase-4 (DPP4), and GLP1-RA drugs that can be used in combination with insulin and glucose-dependent insulinotropic peptide (GIP) agonists, but our research focuses only on the single-component SGLT2i and GLP-1RA.

## Conclusion

In conclusion, this meta-analysis provided evidence that SGLT2i and GLP-1RA in routine clinical care have comparable rates of increased MACE, HHF, MI, and stroke. However, the initiation of SGLT2i versus GLP1-RA was associated with a high occurrence of EKA and GUI and less AKI in elderly person with diabetes. Thus, the judgment of frailty, awareness of adverse events, and dedication of more follow-up time might aid in the care of elderly patients. In addition, there is a need for head-to-head studies with large sample sizes and long-term follow-up periods, especially for elderly patients receiving SGLT2i and GLP-1RA. These studies will help develop appropriate treatment guidelines for older patients with diabetes. Of course, given that SGLT2i and GLP-1RA are currently employed to address conditions beyond diabetes in the elderly population, it is crucial to focus on the application of these two drug classes in non-diabetic elderly individuals in future research. Additionally, the comparison of efficacy and safety between novel hypoglycemic agents with composite components is also worth our attention.

## Data Availability

The original contributions presented in the study are included in the article/**Supplementary Material**. Further inquiries can be directed to the corresponding author.
