# Supplementary material for: Comparative effectiveness and safety of sodium-glucose cotransporter 2 inhibitors vs glucagon-like peptide 1 receptor agonists in elderly patients with type 2 diabetes mellitus: a meta-analysis
Source: Front Endocrinol (Lausanne). 2025 Aug 26;16:1486655. doi: 10.3389/fendo.2025.1486655 (PMC12417164; doi:10.3389/fendo.2025.1486655)

Supplementary table 2. The the funnel plot of publication bias.

MMCE


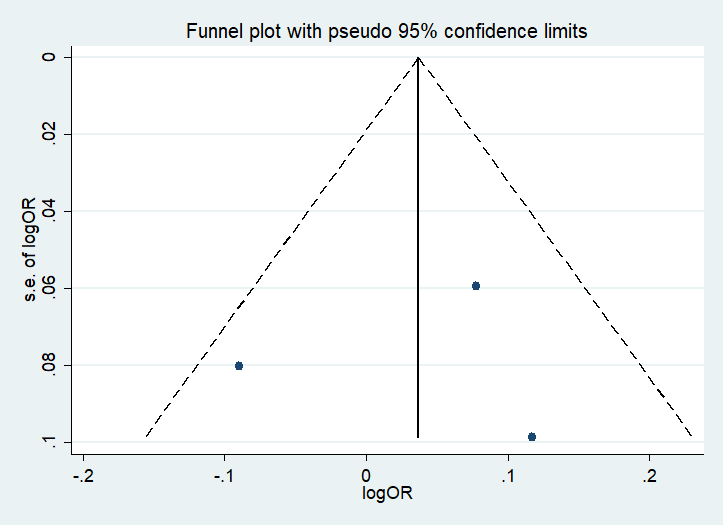


HHF


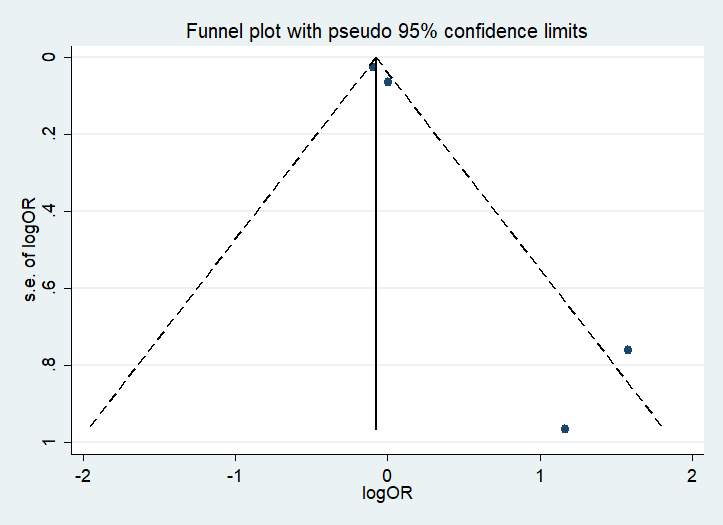


MI


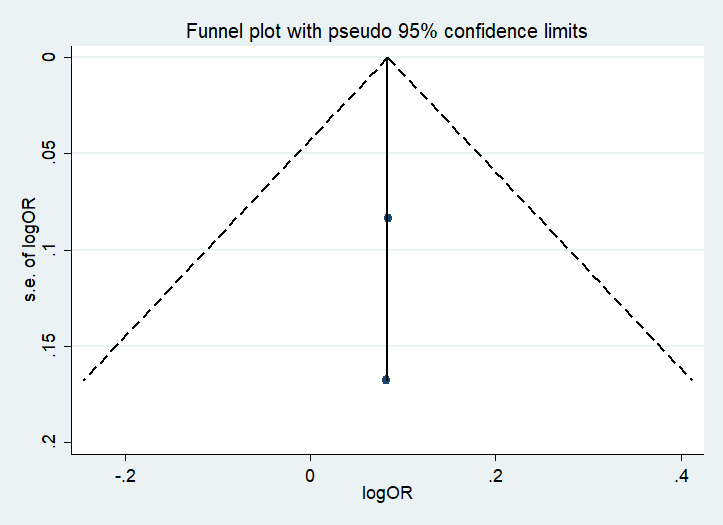


Total AEs


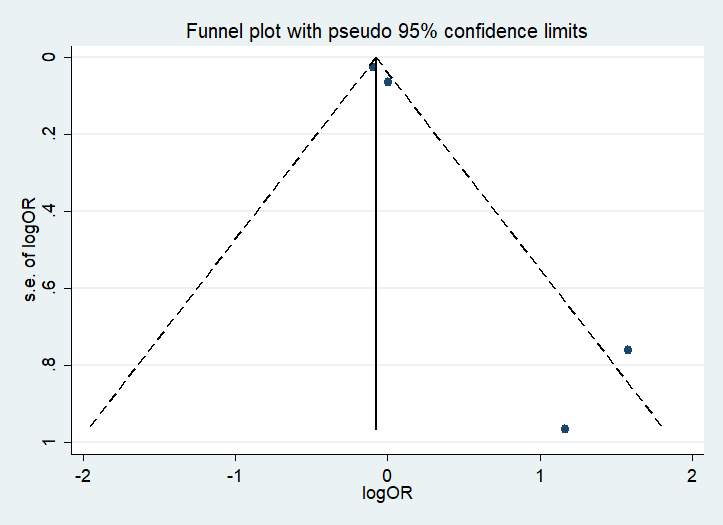


SAEs
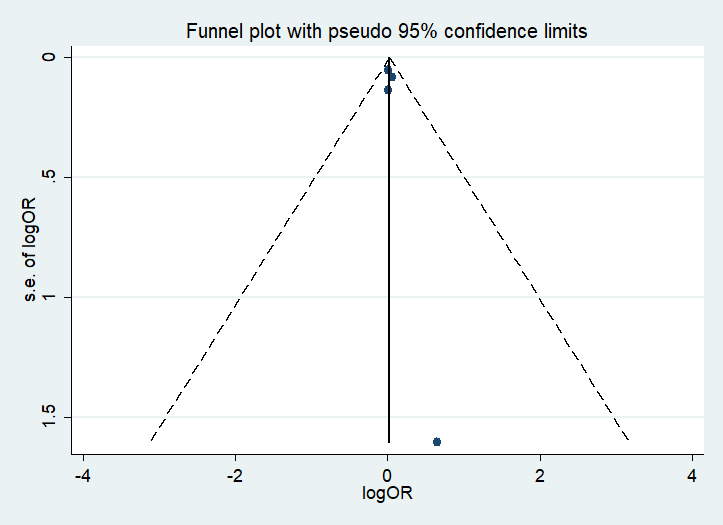


Fractures


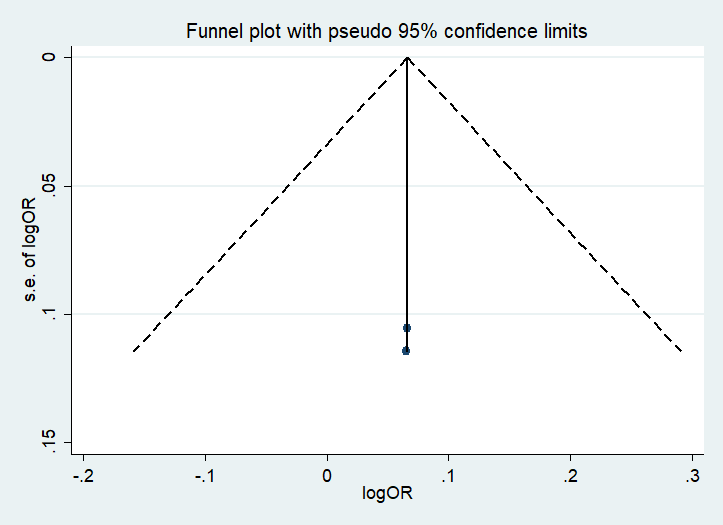


AKI


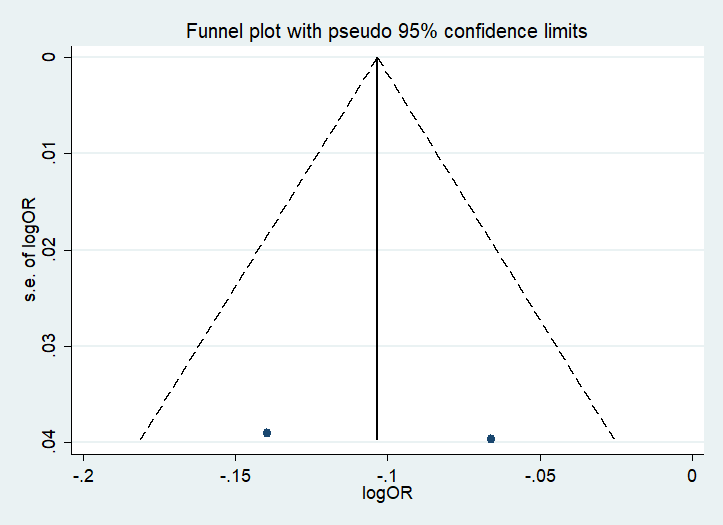


Hypoglycemia


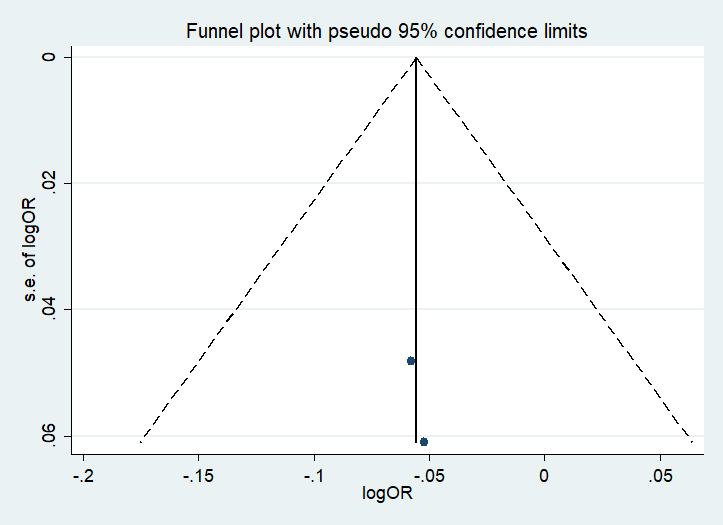


DKA


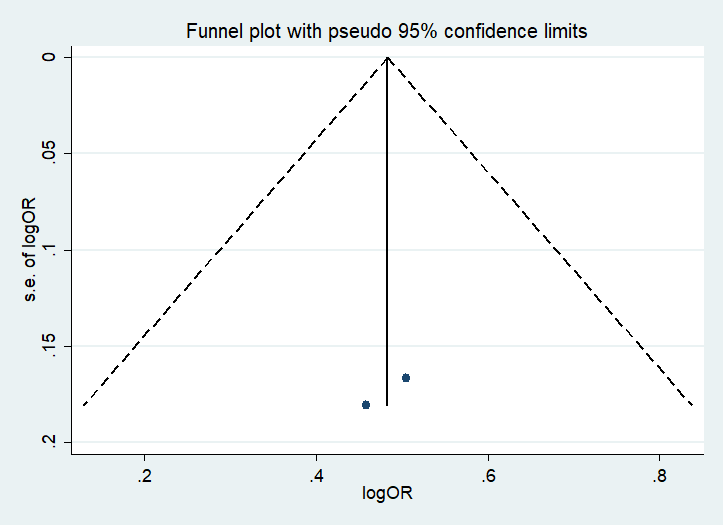


GUIs


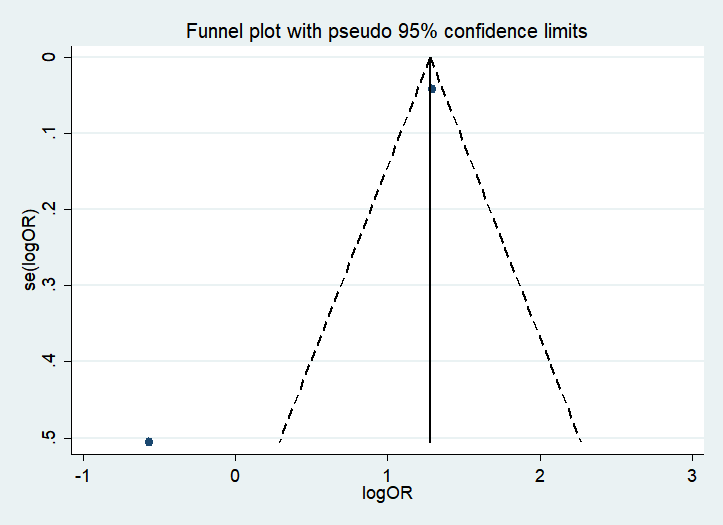

Supplement: Supplementary file 1 [file DataSheet1.docx]
