# Supplementary figures and images for: Comparative effectiveness and safety of sodium-glucose cotransporter 2 inhibitors vs glucagon-like peptide 1 receptor agonists in elderly patients with type 2 diabetes mellitus: a meta-analysis
Source: Front Endocrinol (Lausanne). 2025 Aug 26;16:1486655. doi: 10.3389/fendo.2025.1486655 (PMC12417164; doi:10.3389/fendo.2025.1486655)

Supplementary Table 3. The sensitivity analysis.

MACE


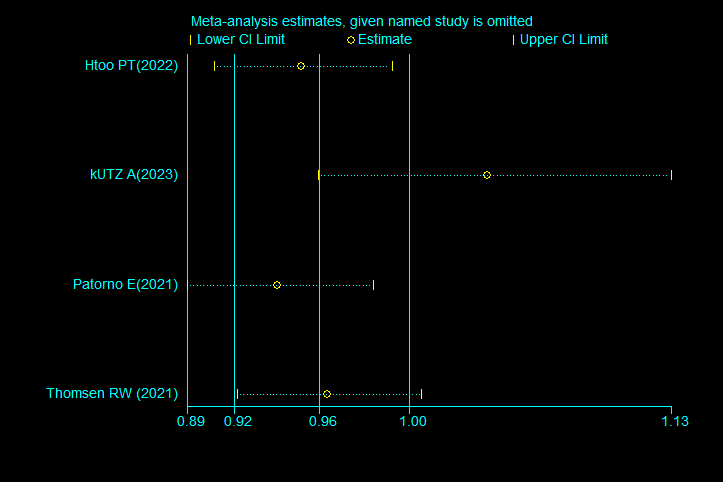


SAEs


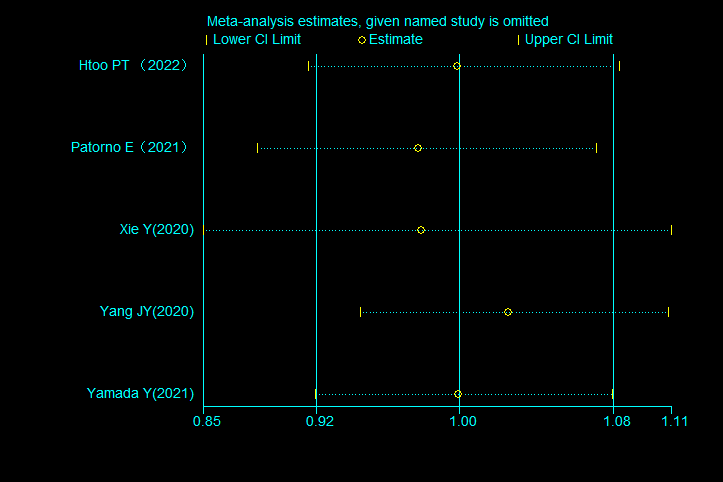

Supplement: Supplementary file 2 [file DataSheet2.docx]
