# Supplementary material for: Comparative effectiveness and safety of sodium-glucose cotransporter 2 inhibitors vs glucagon-like peptide 1 receptor agonists in elderly patients with type 2 diabetes mellitus: a meta-analysis
Source: Front Endocrinol (Lausanne). 2025 Aug 26;16:1486655. doi: 10.3389/fendo.2025.1486655 (PMC12417164; doi:10.3389/fendo.2025.1486655)
Supplement: Supplementary file 3 [file Table1.docx]

| Author | Year | Country | Selection | Comparability | Outcome | Score |
| --- | --- | --- | --- | --- | --- | --- |
| Htoo PT [21] | 2022 | USA | **** | ** | *** | 9 |
| Htoo PT [22] | 2023 | USA | **** | ** | *** | 9 |
| Kutz A [23] | 2023 | USA | **** | ** | *** | 9 |
| Patorno E [14] | 2021 | USA | **** | ** | *** | 9 |
| Thomsen RW [24] | 2021 | Denmark | **** | ** | *** | 9 |
| Varshney N [25] | 2021 | USA | **** | ** | *** | 9 |
| Xie Y [26] | 2020 | USA | **** | ** | *** | 9 |
| Yang JY [27] | 2021 | USA | **** | ** | *** | 9 |
| Yamada Y PIONEER 9 [28] | 2021 | Japan | **** | * | ** | 7 |
| Zhuo M [29] | 2021 | USA | **** | ** | *** | 9 |
| Zhuo M [30] | 2023 | USA | **** | ** | *** | 9 |

Supplementary table 1. The Newcastle-Ottawa Scale.
